# Prevalence and Clustering of Major Cardiovascular Risk Factors in China: A Recent Cross-Sectional Survey

**DOI:** 10.1097/MD.0000000000002712

**Published:** 2016-03-11

**Authors:** Jie Wu, Xinqi Cheng, Ling Qiu, Tao Xu, Guangjin Zhu, Jianhua Han, Liangyu Xia, Xuzhen Qin, Qian Cheng, Qian Liu

**Affiliations:** From the Department of Clinical Laboratory (JW, XC, LQ, JH, LX, XQ, QC, QL), Peking Union Medical College Hospital; Department of Epidemiology and Statistics (TX), Institute of Basic Medical Sciences; and Department of Pathophysiology (GZ), Institute of Basic Medical Sciences, Chinese Academy of Medical Sciences, Peking Union Medical College, Beijing, China.

## Abstract

Cardiovascular disease is the leading cause of death in the Chinese population. Although general prevalence estimates of cardiovascular risk factors (CVRFs) are available for Chinese adults, prevalence estimates covering all adult age groups by race/ethnicity have not been reported. The aim of this study is to estimate the current prevalence and clustering of major CVRFs in Chinese adults, including a plurality of ethnic minorities.

A cross-sectional survey was conducted in a nationally representative sample of 23,010 adults aged 18 years and older from 2007 to 2011. Questionnaires and physical examinations were performed, and fasting blood was collected for laboratory measurements. The prevalence of traditional CVRFs, including hypertension, diabetes, dyslipidemia, overweight, and current smoking, were determined.

The prevalence of the major CVRFs, including hypertension, diabetes, dyslipidemia, overweight, and current smoking were 24.3%, 4.3%, 49.3%, 32.0%, and 21.7%, respectively. These risk factors were significantly associated with sex, age, region, ethnicity, and education levels. Overall, 70.3%, 40.3%, and 16.7% of Chinese adults had ≥1, ≥2, or ≥3 CVRFs, respectively. Men, northern and rural residents were more likely to have clustered CVRFs compared with women, southern and urban residents, respectively. Compared with Han residents, Hui and Mongolian residents were more likely, and Tujia and Miao residents were less likely, to have ≥1, ≥2, or ≥3 risk factors. The prevalence of Chinese women having ≥1, ≥2, or ≥3 CVRFs decreased with increasing levels of education.

The prevalence and clustering of CVRFs is still high in Chinese adults ≥18 years old, especially in men and in individuals living in the northern and rural areas. Of note, there are differences in cardiovascular risk among different ethnic groups. Therefore, targeted and enhanced intervention measures are required to reduce the risk of cardiovascular disease and the corresponding economic burden of disease in China.

## INTRODUCTION

Cardiovascular disease (CVD) has become the most common cause of death in both developed and developing countries worldwide and represents a huge economic burden to humans.^1,2^ Five years ago, the American Heart Association introduced a new concept of “cardiovascular health,” which is defined by 4 cardiovascular health behaviors (smoking, body mass index (BMI), physical activity, and diet) and 4 cardiovascular health factors (total cholesterol (TC), blood pressure (BP), fasting blood glucose (FBG), and smoking), and emphasized health promotion by the prevention of cardiovascular disease risk factors (CVRFs).^3,4^ Hypertension, diabetes, dyslipidemia, overweight/obesity, smoking, and physical inactivity are known to be major risk factors for developing CVD.^5,6^ The clustering of these risk factors in the same individual will increase the incidence of CVD significantly compared with a single risk factor.^7,8^ Although recent data suggest that the prevalence of CVD and CVRFs in some developed countries has been decreasing over time,^5,9–11^ an increased prevalence is still observed in developing countries.^6,12^

In China, due to rapid economic growth and lifestyle changes, the prevalence of CVD and morbidities related to being hypertensive, diabetic, dyslipidemic, and overweight have gradually increased. Recent data suggest that the prevalences of coronary heart disease, stroke, and CVD in adults aged 20 or older have been up to 0.63%, 0.83%, and 1.44%, respectively, and the standardized prevalences in the 2006 population were 0.60%, 0.80%, and 1.37%, respectively.^7^ Recently, several nationally representative population studies have demonstrated that the prevalence and clustering of major CVRFs have increased in China in the past decades.^7,13^ However, all of these studies are based on Han adults and do not include all adult age groups. China is a multiethnic country with 56 ethnic groups. The Han-Chinese account for >90% of the total population, whereas the other 55 ethnic minorities account for <10% of the total population. Among these minorities, 18 ethnic groups, including the Mongolian, Hui, Tibetan, Miao, Yi, Tujia, Korean, Zhuang, Manchu, Uygur, Buyi, Dong, Yao, Bai, Hani, Kazakhs, Dai, and Li populations, comprise 1 million individuals. The geographic distribution of Chinese minorities is extremely uneven, and minority populations living in the northwest and southwest regions of China account for 30.1% and 29.4% of the total minority population, respectively. In China, the minority population is mainly distributed in Inner Mongolia, Xinjiang, Ningxia, Guangxi, Tibet, Yunnan, Heilongjiang, Jilin, Liaoning, Qinghai, Sichuan, Guizhou, Gansu, Hunan, Hubei, and Hainan, and most minorities have specific lifestyles. In addition, racial and ethnic differences are known to influence the prevalence and risks of CVD.^14–16^ Therefore, there is a need to understand the risk factors involved in CVD and to establish rational prevention strategies to decrease morbidity related to CVD in a representative population sample of Chinese multiethnic adults. Currently, no information is available in the literature regarding the ethnic differences in the prevalence of CVD risk factors among Chinese multiethnic adults.

In this study, we used data from a nationally representative sample of multiethnic adults to evaluate the current status of CVRFs and to assess ethnic differences of cardiovascular risk in the general population of China.

## METHODS

### Study Design and Participants

The data used in this study are from the Chinese Physiological Constant and Health Condition (CPCHC) survey, a population-based, cross-sectional survey, which was conducted from 2007 to 2011. This survey used a random, multistage, stratified sampling method to obtain a nationally representative sample of the general Chinese population.^17,18^ Briefly, we selected 6 provinces (Sichuan-Southwest, Yunnan-South, Hunan-South, Inner Mongolia Autonomous Region-North, Ningxia Hui Autonomous Region-Northwest, and Hei Longjiang-Northeast) from different geographical regions (south or north) in China. A 3-stage cluster sampling method was used to select eligible subjects in each province. First, 2 or 3 cities were sampled based on their economic status and the presence of ethnic minorities; then, several communities and villages were randomly selected from each city.

**FIGURE 1:**
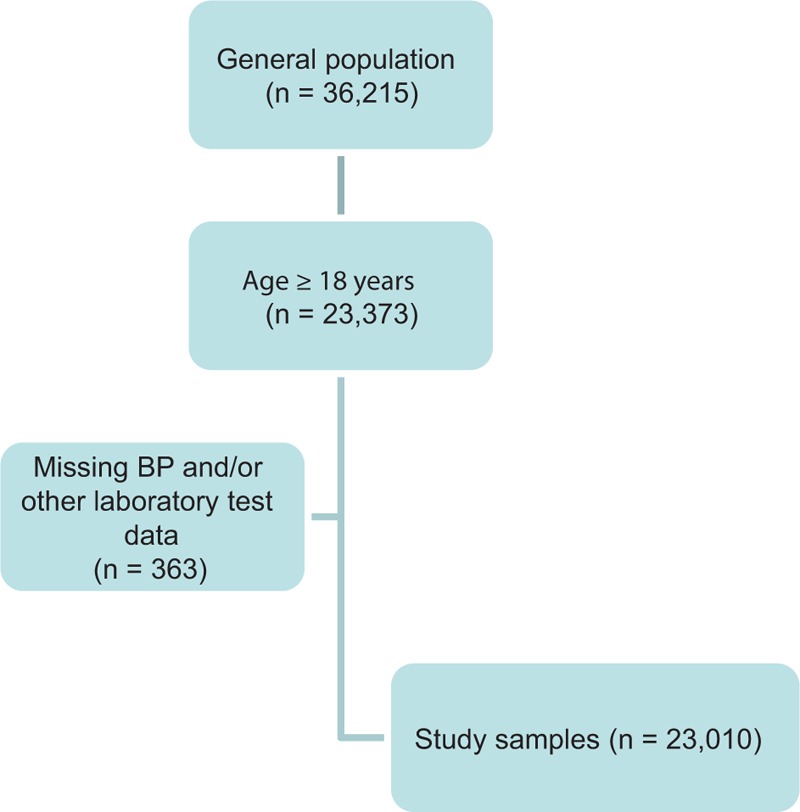
A schematic used for screening and inclusion of the study sample. A total of 36,216 individuals were recruited and had biochemistry measurements collected between 2007 and 2011. Of the 23,373 adults aged ≥18 years, 363 participants had missing data on BP and/or laboratory tests, and as result were excluded. The final sample size was 23,010, which consisted of 10,801 men and 12,209 women. BP = blood pressure.

A total of 36,215 participants were randomly selected to complete the survey and blood biochemical tests after excluding individuals who suffered from systemic disease and confirmed cardiovascular, renal, gastrointestinal, and pulmonary disease or cancer. A schematic of the screening process is presented in Figure 1. Of the total number of participants, 64.5% (n = 23,373) were included after excluding 12,842 subjects aged <18 years. Of the 23,373 adults aged ≥18 years, 363 (1.6%) had missing BP and/or laboratory test data. Therefore, the final sample size of adults included in the present study was 23,010 (10,801 men and 12,209 women), of which 14,744 were Han-Chinese, 2049 were Yi-Chinese, 1909 were Hui-Chinese, 1223 were Mongolian-Chinese, 898 were Korean-Chinese, 775 were Tibetan-Chinese, 658 were Tujia-Chinese, 412 were Miao-Chinese, and 342 were of other ethnicities.

We have performed the calculation of sample size before using these data. According to the existing literatures, we estimated that minimum population prevalence for these risk factors is 5%, and then a minimum sample size should be 7299 if the allowable error is controlled within a range of 0.5%. Actually, the final sample size in this study (23,010 adults, 10,801 men and 12,209 women) fully meets the requirements for minimum sample size. Written informed consent was obtained from each participant prior to data collection. The protocol was approved by the institutional review board of the Institute of Basic Medical Sciences, Chinese Academy of Medical Sciences.

### Questionnaire and Physical Examination

All of the participants were asked to complete a standard questionnaire, which included demographic characteristics (age, sex, ethnicity, address, and physical condition), socioeconomic data (education level, marital status, and occupation), lifestyle risk factors (manual labor, smoking, and drinking status), and their past medical history.

Body weight and height were measured and BMI was calculated as body weight divided by the square of the height (kg/m^2^). Waist circumference (WC) was measured with flexible and inelastic tape at the end of a gentle expiration. Blood pressure was measured using an Omron HEM-7000 electronic sphygmomanometer (Omron Healthcare; Muko, Kyoto, Japan) after the participant had rested for at least 10 minutes.

### Laboratory Measurements

Overnight fasting blood was drawn by venipuncture and all subjects were told to consume a bland diet before blood testing. The blood specimens were centrifuged, and the serum was stored at −80 °C until the laboratory assays were performed. Serum lipids, including TC, triglycerides (TG), high-density lipoprotein cholesterol (HDL-C) and low-density lipoprotein cholesterol (LDL-C), were measured using the Beckman AU Series Automatic Biochemical Analyzer and Sekisui Medical (Japan) reagents. Uric acid (UA), FBG, creatine (Cr), and blood urea nitrogen (BUN) levels were measured using the same instrument and Beckman AU reagents. The biochemical laboratories participating in the survey followed the common internal quality control program, which was standardized by the Peking Union Medical College Hospital.

### Data Interpretation

Hypertension was defined as having an average systolic blood pressure (SBP) ≥140 mm Hg and/or diastolic blood pressure (DBP) ≥90 mm Hg, and/or current antihypertensive medication use. Diabetes was defined as FBG ≥ 7.0 mmol/L and/or current antidiabetes medication use. Dyslipidemia was defined as having at least one of the following: TC ≥ 5.2 mmol/L, TG ≥ 1.7 mmol/L, HDL-C < 1.0 mmol/L, LDL-C ≥ 3.4 mmol/L, and/or current cholesterol-lowering medication use. Overweight/obesity was defined as having a BMI ≥ 25 kg/m^2^. Current cigarette smoking was classified as self-reported responses of “yes” to the question “Do you smoke cigarettes now?”

### Statistical Analysis

The database was constructed with EPI3.02 software by 2 data managers and was corrected to guarantee the accuracy and integration of the data. The data were analyzed using SPSS 16.0 software (SPSS Inc, Chicago, IL). Normally distributed continuous variables (BMI, WC, SBP, DBP, UA, FBG, TC, HDL-C, LDL-C, Cr, and BUN) are presented as the mean ± standard deviation and were analyzed by *t* test. Variables with a skewed distribution (age and TG) are presented as the median (interquartile range) and were compared by the Wilcoxon rank sum test. Categorical data (sex, age group, regions, ethnic group, and education) are presented as percentages and were compared by the χ^2^ test. The tests were performed to compare the variables with the relevant groups. Two-tailed values of *P* < 0.05 were considered statistically significant. The direct standardization method was used for the “age-standardized” prevalence estimates and prevalence estimates of different CVRFs were calculated based on the overall population according to age, in which age-standardized prevalence was determined using the population distribution of China in 2006.^7^ A multivariable logistic regression model was used to explore the association between demographic characteristics and CVD risk factor clustering. We assessed the goodness-of-fit of the logistic regression model using Hosmer–Lemeshow test. For the selection of the explanatory variables, firstly univariate logistic analysis was applied to examine association between each independent variable and clustered risk factors. Then in multivariable logistic regression analyses, all independent variables that were statistically significant and variables that had been shown to have clinical significance were included as potential confounding variables and the adjusted odds ratios (ORs) and 95% confidence intervals were calculated. The data were adjusted for age and sex, and the ORs and 95% confidence intervals were used to assess the risk of CVD risk factor clustering.

## RESULTS

### General Characteristics of the Study Population

As shown in Table 1, the study included 23,010 individuals, 10,801 (46.9%) men and 12,209 (53.1%) women. Nearly one-third of participants were aged 18 to 34 years, and more than half of the population was Han. The ratio of men and women differed by age groups, across different regions, and ethnic groups. The median (interquartile range) age of all participants was 43.0 (30.4–56.3) years old, and no significant difference was observed in the median (interquartile range) age between men and women (*P* = 0.473). Additionally, men had significantly higher BMI, WC, SBP, DBP, UA, FBG, TG, TC, HDL-C, Cr, and BUN levels compared with women (*P* < 0.001).

**TABLE 1:**
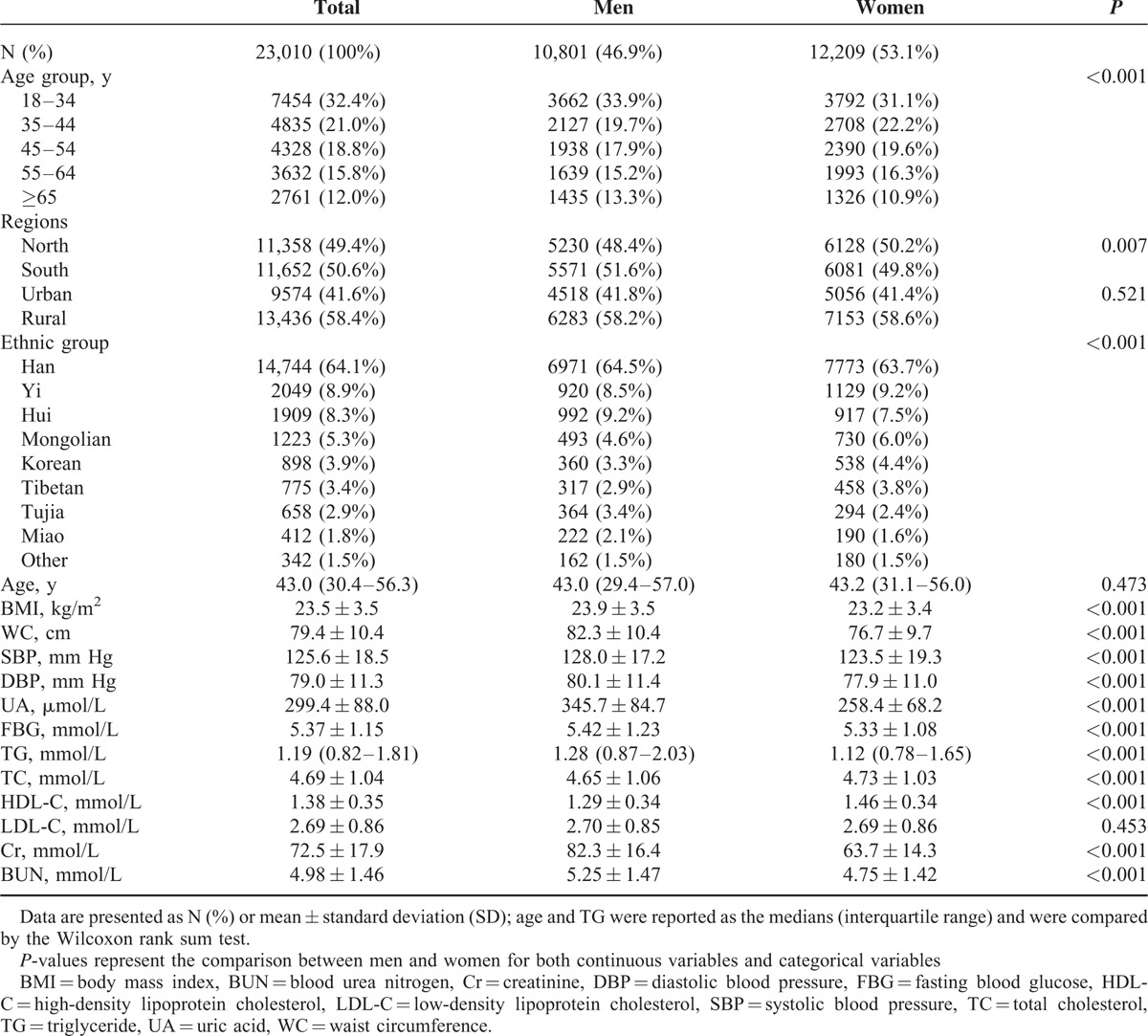
Descriptive Characteristic of the Study Participants

### Prevalence of Major Cardiovascular Disease Risk Factors

Tables 2 and 3 show the age-standardized prevalence of classic CVD risk factors. The overall prevalence of hypertension, diabetes, dyslipidemia, overweight, and current smoking were 24.3%, 4.3%, 49.3%, 32.0%, and 21.7%, respectively. The age-standardized prevalence of these CVD risk factors was higher in men than in women (*P* < 0.001). The prevalence of all of these risk factors increased significantly with increasing age (*P* for trend <0.001), but slightly decreased in participants ≥65 years for dyslipidemia, overweight, and current smoking relative to the total population and regardless of gender (Tables 2 and 3). Except for diabetes, these CVRFs were more common in northern and rural areas compared with southern and urban areas for all people regardless of gender (*P* < 0.01). Notably, there are significant differences in the prevalence of these risk factors among different ethnic groups (*P* < 0.001). The prevalence of hypertension, diabetes, dyslipidemia, overweight, and current smoking was highest in the Hui (36.8%), Korean (4.9%), Yi (61.3%), Mongolian (44.7%), and Miao (31.3%) populations, respectively. Hui, Yi, and Mongolian individuals (of both sexes) had the highest prevalence of hypertension, dyslipidemia, and overweight, respectively. Miao men and Tujia women had the highest rates of diabetes (6.9% and 5.0%, respectively). Miao men and Yi women had the highest rates of current smoking (58.4% and 9.4%, respectively). In the higher education level group, there was a lower prevalence of these CVD risk factors in women, but a higher prevalence of diabetes, dyslipidemia, and overweight in men (Table 3).

**TABLE 2:**
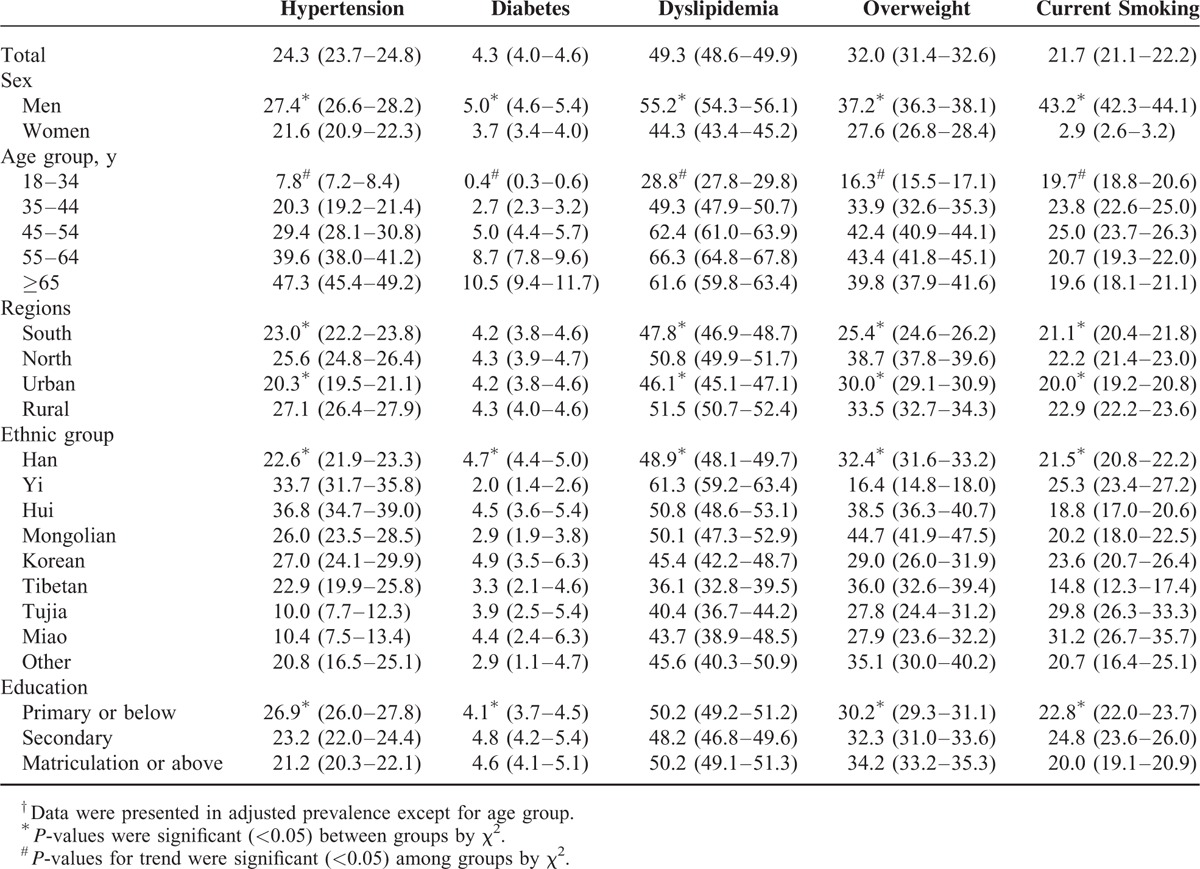
Age-Adjusted^†^ Prevalence of Major Cardiovascular Disease Risk Factors Among Participants

**TABLE 3:**
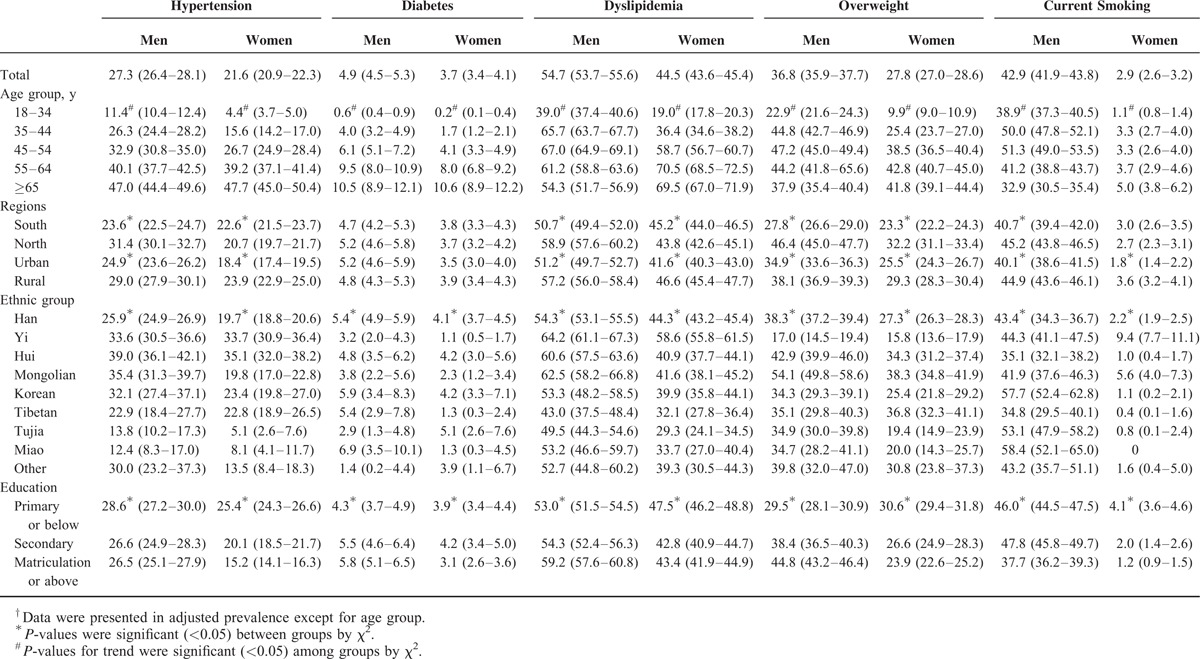
Age-Adjusted^†^ Prevalence of Major Cardiovascular Disease Risk Factors Among Participants by Sex

### Prevalence of Major Cardiovascular Disease Risk Factor Clustering

Overall, the probability of 1 subject having 0, ≥1, ≥2, or ≥3 major CVD risk factors was 29.7%, 70.3%, 40.3%, and 16.7%, respectively, including 18.0%, 82.0%, 52.6%, and 24.4% of men, and 40.1%, 59.9%, 29.4%, and 9.9% of women, respectively (Table 4). The proportion of men who had ≥1, ≥2, or ≥3 risk factors was significantly higher than that of women (*P* < 0.001). With increasing age, the prevalence of CVD risk factor clustering increased within the total sample and in women. However, the proportion of men who had ≥1, ≥2, or ≥3 risk factors was higher among subjects of older age until 55 years old, when the prevalence began to be lower (Table 5). Both in men and women, the prevalence of CVD risk factor clustering, including ≥1, ≥2, or ≥3 risk factors, was higher among northern and rural residents compared with southern and urban residents. The prevalence of ≥1, ≥2, or ≥3 risk factors was highest in the Yi (78.4%), Mongolian (45.5%), and Hui (23.1%) populations relative to the total population, with Korean (89.7%), Mongolian (65.4%), and Mongolian (35.0%) men being at greatest risk, and Yi (71.4%), Yi (36.4%), and Hui (15.6%) women being at greatest risk. In men, there was an obviously increased trend for clustering of ≥2 or ≥3 risk factors with higher education levels. However, in women, there was an obvious decreased trend for the clustering of ≥1, ≥2, or ≥3 risk factors (Tables 4 and 5).

**TABLE 4:**
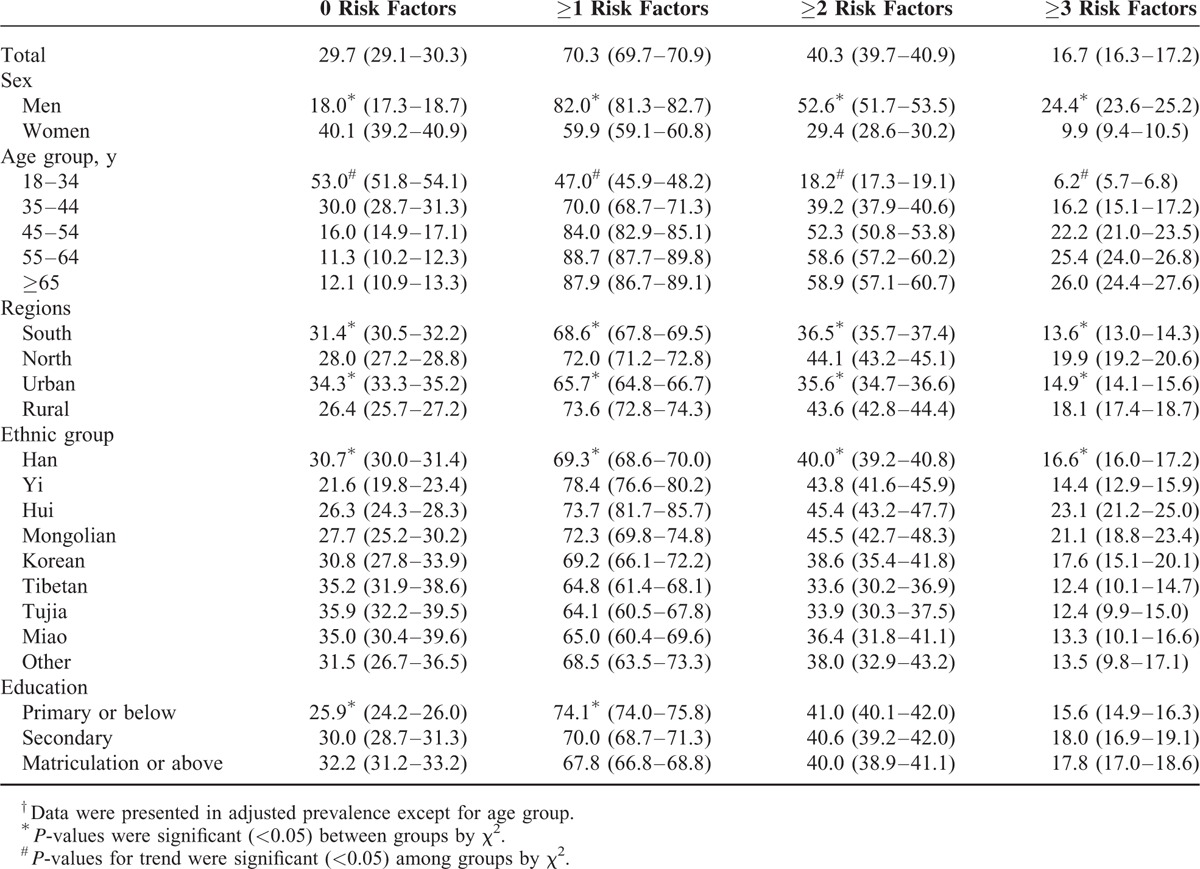
Age-Adjusted^†^ Prevalence of Clustered Cardiovascular Disease Risk Factors Among Participants

**TABLE 5:**
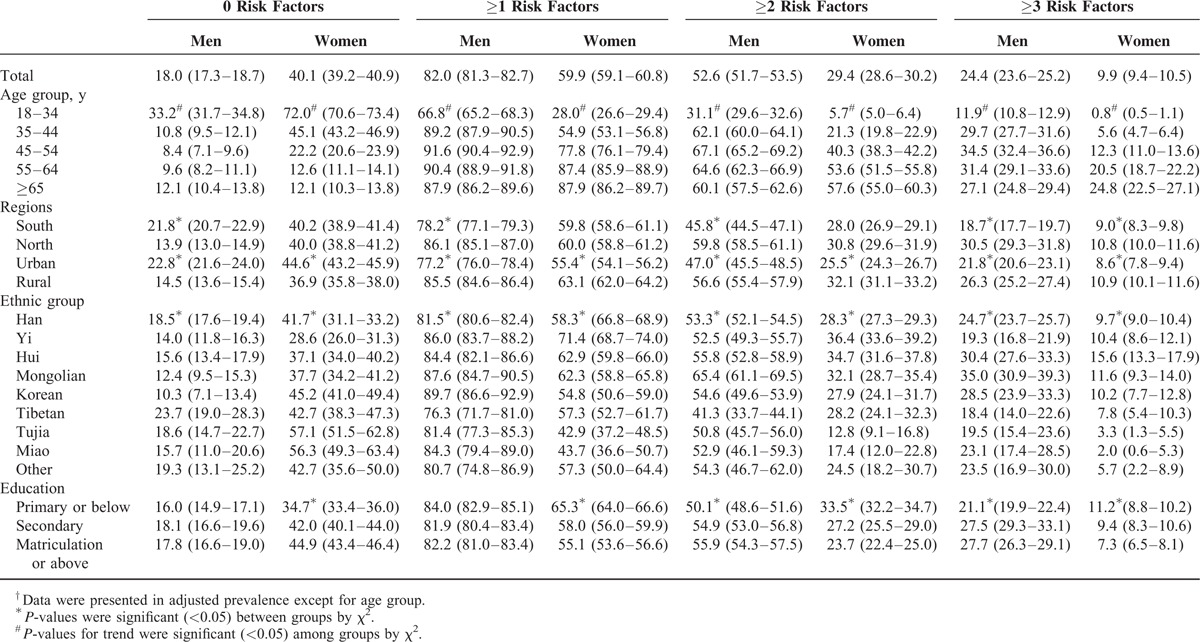
Age-Adjusted^†^ Prevalence of Clustered Cardiovascular Disease Risk Factors Among Participants by Sex

### Multivariable Logistic Regression Analysis

As shown in Table 6, the multivariable logistic regression analysis showed that men, northern and rural residents were more likely to have ≥1, ≥2, or ≥3 risk factors compared with women, southern and urban residents, respectively. Compared with the 18 to 34 years age group, the adjusted OR of having ≥1, ≥2, or ≥3 risk factors increased progressively with increasing age until 65 years, after which it decreased slightly. In the Chinese population aged ≥18 years, Hui and Mongolian residents were more likely and Tujia and Miao residents were less likely to have ≥1, ≥2 or ≥3 risk factors compared with Han residents. The adjusted OR for the prevalence of ≥1 or ≥2 CVD risk factors decreased with increasing levels of education.

**TABLE 6:**
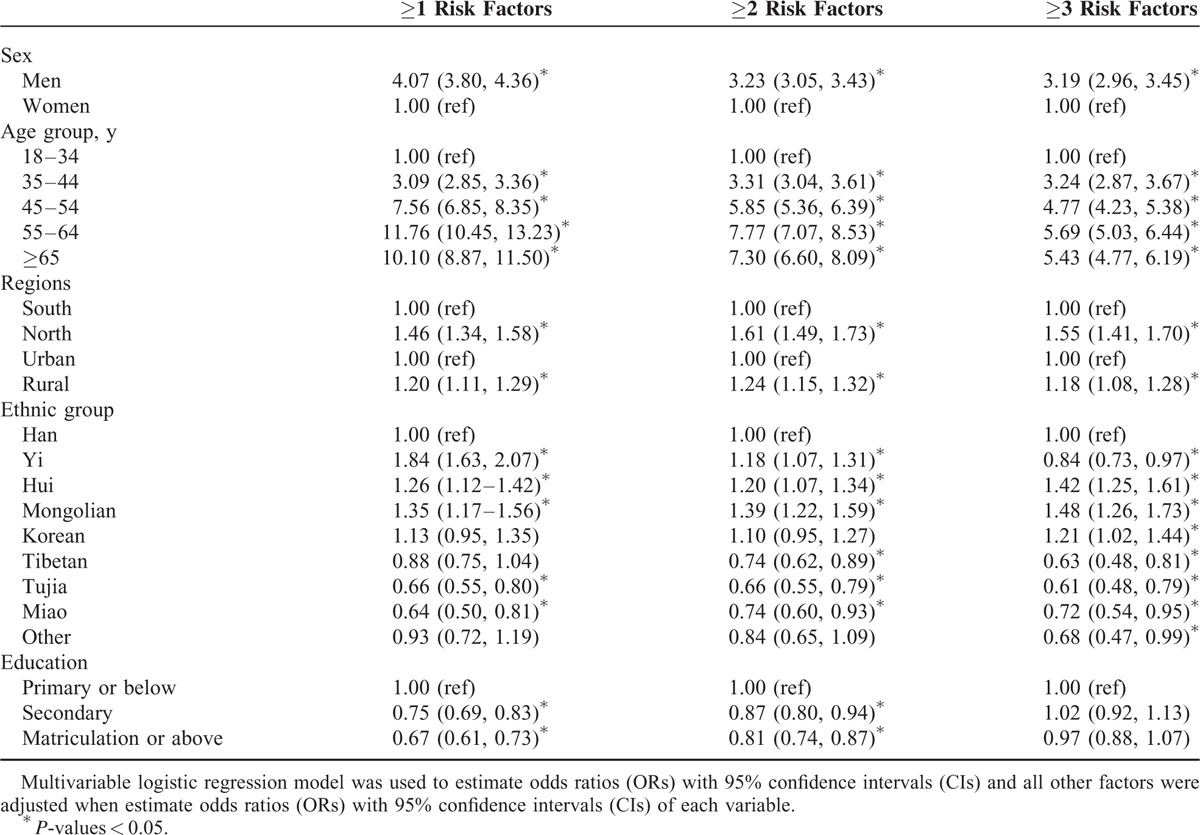
Adjusted Odds Ratio and 95% Confidence Interval of Clustered Risk Factors Associated With Sex, Age, Regions, Ethnic Group, and Education

## DISCUSSION

In this study, we estimated the current prevalence and clustering of major CVRFs among Chinese multiethnic adults and we did not have any hypothesis. To our knowledge, this is the first multicenter study to analyze the prevalence of CVD risk factors and clustering in diverse Chinese provinces, with a large and representative study population covering all adult age groups, and including a plurality of ethnic minorities.

The results from our study showed that 24.3%, 4.3%, 49.3%, 32.0%, and 21.7% adults aged 18 years or over in China have hypertension, diabetes, dyslipidemia, overweight, and current smoking, respectively, which are conventional CVRFs used to predict CVD morbidity and mortality. In 2005, a previous study reported that the prevalence of these CVRFs among Chinese adults aged 35 to 74 years was 26.1%, 5.2%, 53.6%, 28.2%, and 34.4%, respectively.^13^ The slightly lower prevalence of hypertension, diabetes, dyslipidemia, and current smoking in our study may be because a younger age group (18–34 years) was included and the prevalence of most CVRFs increase with age. However, the prevalence of overweight is still higher in our study, which indicates that over the past decade, more people have become overweight/obesity in China and the prevention and control of CVD should emphasize overweight/obesity. In fact, in 2002, 21.8% of Chinese adults aged 18 years and older were overweight or obese based on the WHO standards, and in 1992, the figure was only 14.6%.^19^

Although several studies have demonstrated that the prevalence of some CVRFs is higher in urban populations,^13,20,21^ the data in this study showed that CVRFs, including hypertension, dyslipidemia, overweight, and current smoking, were more common among people living in rural compared with urban areas. This difference may be explained by the rapid urbanization of rural areas^22,23^ and the improvement of the health care system and people's health conditions in urban areas in China during recent years.^24^ A cohort study in New Zealand obtained similar results and showed that rural Maori had significantly higher levels of type 2 diabetes, diagnosed hypertension, treated dyslipidemia, current smoking, and age-adjusted BMI compared with urban Maori.^25^ Another study in China demonstrated that from 1990 to 2003, the mortality rate of CVD increased in rural areas, whereas the figures decreased slightly in urban areas.^19^

A large number of studies have demonstrated that CVD incidence and all-cause mortality increased markedly in the presence of CVRF clustering.^26–28^ Our study revealed that 70.3%, 40.3%, and 16.7% of Chinese adults had ≥1, ≥2, or ≥3 CVRFs, respectively. The significantly higher prevalence of ≥1, ≥2, or ≥3 risk factors among men, northern and rural residents compared with their women, southern and urban counterparts reflects the fact that most CVRFs investigated in this study were higher among men, northern and rural residents. The different patterns between genders, rural–urban areas, and regions are not only a result of the differences in socioeconomic status or economic development but may also be a result of differences in education levels.^29,30^ In this study, we found that the clustering of CVRFs was associated with education levels. Interestingly, in women, there was a decreased risk of clustered CVRFs with higher education levels, but in men, an increased risk of clustered CVRFs was found with higher education levels, which is likely due to differences in occupations, family responsibilities, and health concerns. Although highly educated people tend to have more health awareness, the gender-specific conception of health may be influenced by education levels.^31^ Highly educated women often pay more attention to their body shape and health and consider thinness to be desirable. In contrast, highly educated men attend more social occasions and eat out more frequently (including drinking and overeating high-fat and high-energy foods), which easily causes diabetes, dyslipidemia, and overweight. In this study, we also found that, in men, the CVRFs increased with increasing age up to the age of 55 years, but decreased thereafter. A possible reason for this observation is as follows: younger men tend to have adverse eating and lifestyle habits (eg, preferring wine, a high-fat diet, more social intercourse, and staying up late). However, older men gradually pay more attention to their own physical health because they have more time and are more worried about health after retirement.

Differences in the prevalence of CVD and associated risk factors have been noted across ethnic groups both within and between countries.^14,15,32,33^ China is a multiethnic country, and the different ethnic groups have specific dietary habits and lifestyles, which may affect their cardiovascular health status. In this study, the population was ethnically diverse, and the prevalence of CVRFs was significantly different between ethnicities. Compared with Han residents, Hui and Mongolian residents were more likely, and Tujia and Miao residents were less likely, to have ≥1, ≥2, or ≥3 risk factors. A recent study reported that hypertension prevalence in ethnic Hui and Mongolian minorities was significantly higher than in the Han population,^34^ which is consistent with our findings. Notable differences exist in the CVRF assessment by ethnicity, region, and education that require central and local governments to develop related policies and guidelines to improve the health care system and strengthen interventions.

A major strength of the present study is that it is a population-based study with a representative sample of the general Chinese adult population, including a plurality of ethnic minorities. Additionally, the larger sample size ensures sufficient power in estimating the prevalence and clustering of major CVRFs, as well as determining the association between clustered risk factors and sex, age, regions, ethnic group, and education.

The study also has several limitations. First, the study is based on a cross-sectional survey, and thus cannot determine causality or the temporal relationship between CVRF clustering and the incidence of CVD. However, previous studies have demonstrated the importance of CVRF clustering to the incidence of CVD. Second, the population sizes of some minorities were relatively small because of the limited ethnic group population in the study regions compared to the Han population; however, a 4-stage randomly stratified cluster sampling method was used to make the study population representative. Third, we assessed these major CVRFs according to the commonly used international standards and did not have physician diagnoses for diseases, although a history of self-reported disease was included. Last, there are geographical variations in the distribution of ethnic minorities in China and we also found the significant differences in the prevalence of CVRFs between ethnicities, so the sampling design may cause the presence of some degree of intracluster correlation, which may be a major limitation of the study.

In conclusion, our data highlight the high prevalence and clustering of CVRFs in Chinese adults, especially among some minority groups such as the Hui and Mongolian populations. A healthy lifestyle, such as weight control, the avoidance or cessation of smoking, eating a healthy diet, and undertaking regular physical activities should be emphasized not only in the Han population but also in minority ethnic groups. Corresponding public health programs are required to improve this situation in the Chinese population.
